# Comprehensive Analysis and Characterization of *Mangifera indica* L. Leaf Powder

**DOI:** 10.1002/fsn3.70083

**Published:** 2025-06-05

**Authors:** Jasjit Kaur, Deepika Kaushik, Mukul Kumar, Gülçin Emel Babagil, Ryszard Amarowicz, Charalampos Proestos, Emel Oz, Fatih Oz, Tuba Esatbeyoglu, Matteo Bordiga

**Affiliations:** ^1^ Department of Food Technology and Nutrition Lovely Professional University Phagwara Punjab India; ^2^ Department of Biotechnology, Faculty of Applied Sciences and Biotechnology Shoolini University Solan Himachal Pradesh India; ^3^ Republic of Türkiye Ministry of Agriculture and Forestry Erzurum Directorate of Provincial Agriculture and Forestry Erzurum Türkiye; ^4^ Institute of Animal Reproduction and Food Research Polish Academy of Sciences Olsztyn Poland; ^5^ Laboratory of Food Chemistry, Department of Chemistry, School of Sciences National and Kapodistrian University of Athens Athens Greece; ^6^ Department of Food Engineering, Agriculture Faculty Ataturk University Erzurum Türkiye; ^7^ Engineering Faculty, Department of Food Engineering Krygyz‐Turkish Manas University Bishkek Kyrgyzstan; ^8^ Department of Molecular Food Chemistry and Food Development, Institute of Food and One Health Gottfried Wilhelm Leibniz University Hannover Hannover Germany; ^9^ Department of Pharmaceutical Sciences Università degli Studi del Piemonte Orientale “A. Avogadro” Novara Italy

**Keywords:** FESEM, FTIR, leaf, *Mangifera indica*, techno‐functional, TGA, XRD

## Abstract

*Mangifera indica* L., commonly known as mango, is a tropical evergreen tree that belongs to the Anacardiaceae family and shows a number of biological properties, such as anticancer, anti‐inflammatory, antioxidant, antidiabetic, and antibacterial activities. In the present study, the proximate composition, techno‐functional properties, as well as characterization of *Mangifera indica* L. leaf powder using FTIR (Fourier transform infrared spectroscopy), TGA (thermal gravimetric analysis), XRD (X‐ray diffraction), and FESEM (field emission scanning electron microscopy), were discussed. The proximate analysis included moisture (11.46% ± 0.50%), ash (7.56% ± 0.40%), fat (4.53% ± 0.35%), crude fiber (8.50% ± 0.30%), protein (18.16% ± 0.76%), and carbohydrates (49.76% ± 0.30%). In the techno‐functional properties, the bulk density (0.39 ± 0.001 g/cm^3^), tapped density (0.45 ± 0.004 g/cm^3^), Carr's index (12.40 ± 0.47), Hausner ratio (1.14 ± 0.01), water absorption index (3.00 ± 0.05 g/g), water solubility index (19.80% ± 0.30%), foaming capacity (32.07% ± 0.52%), foam stability (21.86% ± 0.43%), swelling capacity (9.06 ± 0.61 mL/g), and oil absorption capacity (3.67 ± 0.05 g/g) were determined. The FTIR analysis revealed the nature of various functional groups present in the powder, while the TGA determined the thermal stability of the powder. The amorphous or crystalline form of the powder was revealed using the XRD technique, and the surface morphology was analyzed using FESEM. This study offers an extensive understanding of the techno‐functional properties and characterization of the *Mangifera indica* L. leaf powder, which demonstrates its multifaceted properties and potential to be used as a promising ingredient for the development of formulations and value‐added products in industries.

## Graphical abstract

*Mangifera indica* L., a tropical evergreen tree with numerous biological properties, was studied for its proximate composition, techno‐functional properties, and characterization. The study found that the mango leaf powder had good water absorption, solubility index, foaming capacity, swelling capacity, and oil absorption capacity. The powder's amorphous or crystalline form and surface morphology suggest it could be a promising ingredient for industry formulations and value‐added products.

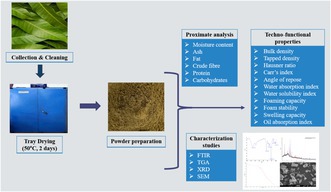

## 1 Introduction

Mango ( *Mangifera indica* L.) has been the topic of comprehensive research to study biomolecules from its various parts, including leaves, stems, seeds, and fruits (Shah et al. 2025; Nwozo et al. 2023). It belongs to the Anacardiaceae family and is considered a historically and economically significant tropical fruit crop worldwide. Apart from its well‐known fruits, *Mangifera indica* L. is a tropical evergreen tree that has a number of medicinal properties (El‐Nashar et al. 2024; Kumar et al. 2021). Its therapeutic value is widely documented, and it has been utilized for decades to treat various types of diseases. Various pharmacological activities, including anti‐inflammatory, anticancer, antidiabetic, and antibacterial properties, have been demonstrated by this plant (Mehmood et al. 2024; El‐Nashar et al. 2024). The mango is indigenous to South and Southeast Asia, and its leading producers are China, India, Mexico, Thailand, Indonesia, Brazil, Bangladesh, Pakistan, Philippines, Egypt, and Nigeria (Ahmad et al. 2023; Rajan and Hudedamani 2019). Due to the presence of essential nutrients, every part of the mango tree, such as fruits, flowers, leaves, stems, seeds, pulp, and peel, can be utilized (Quintana et al. 2021). Mango leaves are an essential source of crude protein, dietary fiber, minerals, vitamins, and bioactive molecules like phenolics as well as essential oils. Protein is the major macromolecule found in mango leaves. Minerals like nitrogen, potassium, phosphorus, magnesium, calcium, sodium, sulfur, iron, and vitamins (A, B, C, E) are abundant in mango leaves (Ali et al. 2020; Princwill‐Ogbonna et al. 2019). To help with the livestock food shortage in underdeveloped nations, mango leaves can be used as an alternative feed source for animals (Kumar et al. 2021).

Various studies have identified the presence of phenolic compounds, tannins, saponins, flavonoids, steroids, alkaloids, xanthones, terpenoids, anthraquinones, and glycosides in mango leaves (Chisom et al. 2023; Jhaumeer Laulloo et al. 2018). Most abundant are total phenolic compounds (TPCs), such as flavonoids, phenolic acids, terpenoids, tannins, xanthones, and benzophenones. The effectiveness of TPCs against various chronic diseases, including diabetes, cancer, neurological disorders, and cardiovascular disorders, has been demonstrated by numerous epidemiological studies (Mehmood et al. 2024; Rasouli et al. 2017). TPCs regulate various physiological processes, including enzymatic activity, cellular redox potential, cell proliferation, and signal transduction pathways to prevent chronic diseases (Luca et al. 2020). Mangiferin is the most active biological component of mango leaves among all TPCs, followed by phenolic acids, antioxidants (carotenoids, flavonoids, quercetin, ascorbic acid, tocopherols, and isoquercetin), and benzophenones (Kumar et al. 2021). It is the major and significant component in the mango leaf extract. It is a naturally occurring polyphenol and an antioxidant that helps to reduce cholesterol formation, regulate glucose metabolism, improve insulin resistance, and suppress tumor necrosis factor α expression, as well as inducible nitric oxide synthase enzyme. The majority of the biological activities of mango leaves are mainly attributed to mangiferin (Pan et al. 2018; Swaroop et al. 2018).

Mango leaves have a wide range of applications due to their diverse phytochemical and biological activities, including antimicrobial, antidiabetic, antioxidant, anti‐inflammatory, antiobesity, antitumor, and immunomodulatory actions (Prakash et al. 2023; Mirza et al. 2021). Mango leaves also contain benzophenone compounds, which show significant antioxidant, immunosuppressive, and α‐glucosidase inhibitory properties (Gu et al. 2019). Compounds like monoterpenes, sesquiterpenes, and trace levels of oxygenated and nonterpenoid hydrocarbons are found in mango leaf oil. The chemical compounds in mango leaf oil, such as camphor, α‐humulene, α‐gurjunene, α‐selinene, and trans‐caryophyllene, possess bacteriostatic and antimicrobial properties (Mehmood et al. 2024; Fontenelle et al. 2017). Due to their antioxidant and anti‐inflammatory properties, polyphenols in mango leaves demonstrate potent antiproliferative effects against various types of cancers, including breast, pancreatic, colon carcinoma, and others (Ahmad et al. 2023; Yap et al. 2021). The bioactive xanthone mangiferin mainly contributes to the antitumor properties of mango leaf extracts (Klein‐Júnior et al. 2020). Mango leaf extracts show hepatoprotective activity (Ramírez et al. 2018) and have an essential role as antiobesity agents (Sandoval‐Gallegos et al. 2018). These extracts have been used as traditional medicines to treat asthma, diabetes, diarrhea, bronchitis, respiratory and liver problems, scabies, syphilis, and kidney disorders (Kumar et al. 2021).

This study aimed to determine the proximate estimation (moisture content, protein, ash, fat, crude fiber, carbohydrates) and techno‐functional properties (bulk density, tapped density, Hausner ratio, Carr's index, angle of repose, water absorption index, water solubility index, swelling capacity, foam stability, foaming capacity, oil absorption capacity) of the *Mangifera indica* L. leaf powder, and its characterization was done using various techniques such as FTIR (Fourier transform infrared spectroscopy), TGA (thermal gravimetric analysis), XRD (X‐ray diffraction), and FESEM (field emission scanning electron microscopy).

## 2 Materials and Methods

### 2.1 Materials

All the primary chemicals of analytical grade were used, and the preparation of reagents was done in class A‐certified glassware. Chemicals such as sodium hydroxide, sulfuric acid, hexane, copper sulfate, potassium sulfate, and boric acid were procured from Loba Chemie Pvt. Ltd. Mumbai, Maharashtra, India.

### 2.2 Collection, Cleaning, and Drying of *Mangifera indica* L. Leaves

The leaves of *Mangifera indica* L. were gathered from the agricultural field of Lovely Professional University, Phagwara, Punjab, India. The collected leaves were cleaned a few times with tap water, followed by distilled water. After two days of drying at 50°C in a tray dryer (PPI FiniX72, PPI Projects PVT. Limited, Delhi, India), the leaves were powdered with a grinder (Sujata Powermatic plus 900 watts) and then stored in a plastic bag (Ziploc) for further study.

### 2.3 Proximate Analysis of *Mangifera indica* L. Leaf Powder

#### 2.3.1 Moisture Content

The moisture content of *Mangifera indica* L. leaf powder was calculated using a method given by Dey et al. (2025). The sample powder (5 g) was taken in preweighted Petri dishes and kept in a preheated hot air oven (Oven universal NSW‐143) at 105°C. After every 1 h, Petri plates were transferred to a desiccator (Borosil) to cool down, and their weight was measured till constant weight was obtained.

$$\text{Moisture}\;\left(\%\right)=\frac{\left(W_{1}-W_{2}\right)}{W_{S}}\times 100$$

where *W*_1_ = initial weight of Petri plates and the sample (g); *W*_2_ = final weight of Petri plates and the sample after drying (g); *W*_*S*_ = weight of the sample (g).

#### 2.3.2 Determination of Ash Content

The ash content of *Mangifera indica* L. leaf powder was calculated using a method provided by Aniqa and Rizvi (2025). The sample (2 g) was taken into a clean, dry, and preweighed crucible, charred on a burner till no fumes were observed. The crucible was placed in a muffle furnace (NSW 101, Narang Scientific Works Pvt. Ltd. New Delhi, India) and heated at 550°C for six hours, and its weight was measured after being cooled in a desiccator. The ash content of the sample was determined using the formula:

$$\mathrm{Ash}\;\left(\%\right)=\frac{\left(W_{1}-W_{2}\right)}{W_{S}}\times 100$$

where *W*_1_ = Wt. of crucible and ash (g); *W*_2_ = Wt. of empty crucible (g); and *W*_*S*_ = Wt. of the sample (g).

#### 2.3.3 Determination of Fat Content

The fat content of *Mangifera indica* L. leaf powder was measured according to the method given by Irudaya et al. (2023). The sample (2 g) taken in a thimble was extracted with hexane as a solvent in a Soxhlet extractor (Socs plus, pelican INC Chennai, India) for 1.5 h at 100°C. The final weight was obtained by evaporating the solvent at 80°C–90°C. The fat content of the sample was calculated using the formula:

$$\text{Crude}\;\mathrm{fat}\;\left(\%\right)=\frac{\left(W_{1}-W_{2}\right)}{W_{S}}\times 100$$

where *W*_1_ = Wt. of flask after evaporation (g); *W*_2_ = Initial wt. of the empty flask (g); and *W*_*S*_ = Wt. of the sample (g).

#### 2.3.4 Determination of Crude Fiber

The crude fiber content of the mango leaf powder was estimated using the method given by Fufa et al. (2025). The 2 g of defatted sample and 200 mL of 1.25% H_2_SO_4_ were boiled in a digestion flask for 30 min. The residue was then filtered through filter paper (Whatman No. 1) and washed with boiling water to remove any remaining acid. Further, it was boiled for 30 min with 200 mL of 1.25% NaOH, filtered through muslin cloth, and then with hot water until it became alkali‐free. The residue was then taken in a preweighed crucible and it was dried at 100°C in a hot air oven till constant weight was obtained. The dried residue was charred on a burner and kept in a muffle furnace at 550°C–600°C for three hours, and weight was obtained after being cooled in a desiccator. The given formula determined the sample's crude fiber content:

$$\text{Crude}\;\text{fiber}\;\left(\%\right)=\frac{\left(W_{1}-W_{2}\right)}{W_{S}}\times 100$$

where *W*_1_ = Wt. of residue (g); *W*_2_ = Wt. of ash (g); and *W*_*S*_ = Wt. of the sample (g).

#### 2.3.5 Determination of Protein Content

The protein content of mango leaf powder was estimated using the Kjeldahl apparatus (Kjeloplus, pelican INC Chennai, India) (Irudaya et al. 2023). The sample (0.3 g) was taken in a digestion flask, followed by a catalyst mixture (K_2_SO_4_ and CuSO_4_) in the ratio of 5:1 and 10 mL of conc. H_2_SO_4_. After that, the mixture was digested for 3–4 h on a digestion heater at 420°C until the appearance of a clear solution. After removing the flask and allowing it to cool, the digested sample was transferred into a flask and diluted with deionized water (100 mL). 10 mL of 40% NaOH was used to neutralize 5 mL of the sample. The distillate was then poured into a conical flask with 2% boric acid (10 mL) and one drop of indicator, and it was titrated against 0.1 N H_2_SO_4_ to obtain the endpoint (red color). The given formula determined the protein content of the sample:

$$\text{Nitrogen}\;\left(\%\right)=\frac{T\times 0.1\times 0.014\times 100}{W_{S}}$$

where *T* = titration value; 0.1 = normality of H_2_SO_4_; 0.014 = atomic wt. of nitrogen/1000; *W*_*S*_ = Wt. of the sample

$$\text{Protein}\;\left(\%\right)=\text{Nitrogen}\;\left(\%\right)\times 6.25$$

#### 2.3.6 Carbohydrate Content Determination

Carbohydrate content was calculated by the formula given by Jaglan et al. (2023).

$$\text{Carbohydrates}=100-\%\left(\text{Moisture}+\mathrm{Fat}+\mathrm{Ash}+\text{Fiber}+\text{Protein}\right)$$

### 2.4 Techno‐Functional Properties of *Mangifera indica* L. Leaf Powder

The dried *Mangifera indica* L. leaf powder was used for the techno‐functional analysis.

#### 2.4.1 Bulk Density, Tapped Density, Carr's Index, and Hausner Ratio

The bulk density (BD) of the sample was calculated using the formula (Vega‐Castro et al. 2025):

$$\text{Bulk}\;\text{density}\;\left(g/{\mathrm{cm}}^{3}\right)=\frac{W_{1}-W_{2}}{V}$$

where *W*_1_ = Wt. of the sample + container (g), *W*_2_ = Wt. of the empty container (g), *V* = Vol. of the container.

The tapped density (TD), Carr's index, and Hausner ratio were estimated using the method given by Awari et al. (2024) and the formula given below:

$$\text{Tapped density}\;\left(g/{\mathrm{cm}}^{3}\right)=\frac{M}{V}$$

where *M* = powder mass (g), and *V* = final tapped vol.

Carr's index (CI) determines the flowability of the powder and is estimated using the formula:

$${\text{Carr}}^{'}s\;\text{index}=\frac{\mathrm{TD}-\mathrm{BD}}{\mathrm{TD}}\times 100$$

The Hausner ratio (HR) represents the compressibility of powder or any granular material and is calculated by the formula:

$$\text{Hausner ratio}=\frac{\mathrm{TD}}{\mathrm{BD}}$$

#### 2.4.2 Angle of Repose

The heap's height (*H*) and the diameter (*D*) of mango leaf powder were obtained, and the angle of repose (*φ*) was determined using the given formula (Meghwal et al. 2024):

$$\varphi =\tan^{-1}\;\frac{2H}{D}$$

#### 2.4.3 Water Absorption Index (WAI) and Water Solubility Index (WSI)

The WAI and WSI were calculated using the method described by Bruttomesso et al. (2024) with minor modification. The 3 g of *Mangifera indica* L. leaf powder in 30 mL distilled water was kept at 30°C for 30 min in the water bath and centrifuged for 10 min at 2490 *g*. The supernatant was then collected and used to calculate WAI and WSI by using the formula:

$$\mathrm{WAI}\;\left(g/g\right)=\frac{\mathrm{Wt}.\;\text{of hydrated residue}}{\mathrm{Dry}\;\text{sample}\;\mathrm{wt}.}$$

$$\mathrm{WSI}\;\left(\%\right)=\frac{\mathrm{Wt}.\;\text{of dissolved solids in supernatant}}{\mathrm{Dry}\;\text{sample}\;\mathrm{wt}.}\times 100$$

#### 2.4.4 Foaming Capacity and Stability

Foaming capacity and stability were estimated by the method described by Amiri et al. (2024) with slight modification. The 2 g of *Mangifera indica* L. leaf powder in 100 mL of distilled water was whipped at high speed for a few mins. The sample was then poured into a graduated cylinder (250 mL), and the total foam volume after whipping was calculated.

$$\text{Foaming capacity}\;\left(\%\right)=\frac{\mathrm{Vol}.\;\text{after whipping}-\mathrm{Vol}.\;\text{before whipping}}{\mathrm{Vol}.\;\text{before whipping}}\times 100$$

$$\text{Foam stability}\;\left(\%\right)=\frac{\text{Foam}\;\mathrm{vol}.\;\text{after}\;1\;h}{\text{Initial foam}\;\mathrm{vol}.}\times 100$$

#### 2.4.5 Swelling Capacity

Swelling capacity (SC) was calculated using the method given by Pineda‐Vargas et al. (2020). A graduated cylinder (50 mL) was filled with 2.5 g of *Mangifera indica* L. leaf powder, and 30 mL of distilled water was poured into it. To achieve hydration, the filled cylinder was kept for 16 h, and the final volume absorbed by the sample was obtained. The swelling capacity was measured using the formula:

$$\mathrm{SC}\;\left(\mathrm{mL}/g\right)=\frac{\text{Final}\;\mathrm{vol}.}{\text{Sample}\;\mathrm{wt}.}$$

#### 2.4.6 Oil Absorption Capacity (OAC)

OAC was calculated according to the method proposed by Singh, Inbaraj, et al. (2022) and Singh, Rasane, et al. (2022). The 0.3 g of *Mangifera indica* L. leaf powder in 3 mL of soybean oil was taken in a graduated centrifuge tube (10 mL), centrifuged at 542 *g* for 30 min, and the supernatant was eliminated. After re‐weighing the tubes, the OAC was determined by the formula:

$$\mathrm{OAC}\;\left(g/g\right)=\frac{\mathrm{Wt}.\;\text{of sample}+\text{oil}}{\text{Sample}\;\mathrm{wt}.}$$

### 2.5 Characterization of *Mangifera indica* L. Leaf Powder

#### 2.5.1 FTIR (Fourier Transform Infrared) Analysis

FTIR analysis of the *Mangifera indica* L. leaf powder was performed using a Perkin Elmer FTIR Spectrometer (PerkinElmer Inc. USA) with ATR (attenuated total reflectance) and pellet accessories. A total of 5 mg of sample was utilized for analysis; the spectra were observed in the mid‐infrared region (4000–400 cm^−1^), and the data were recorded with the help of Spectrum 10 software (Nan et al. 2025).

#### 2.5.2 TGA (Thermal Gravimetric Analysis)

Perkin Elmer TGA 4000 was utilized to study the thermal stability of the *Mangifera indica* L. leaf powder. 2.4 mg of sample was first heated to the initial temperature of 30°C and then raised to 600°C at a heating rate of 10°C/min. Nitrogen was used as the carrier gas with a rate of 20 mL/min. At 600°C, the sample was held for a minute, then the weight changes were observed, and the values were recorded (Souza et al. 2025).

#### 2.5.3 XRD (X‐ray Diffraction)

X‐ray diffractometer (X'Pert PRO, PANalytical, Almelo, Netherlands) was employed to produce the diffraction pattern of the *Mangifera indica* L. leaf powder using a Cu‐based anode X‐ray tube. The sample was placed in a sample holder within an X‐ray chamber with Cu Kα radiation (*λ* = 0.15406 nm), and the spectra were recorded at a scanning rate of 10°/min, using a diffraction angle range of 10°–50° (2θ), operated at 30 mA and 40 kV voltage (Mousa et al. 2024).

#### 2.5.4 FESEM (Field Emission Scanning Electron Microscopy)

FESEM (Hitachi Se 300H‐Tokyo, Japan) was utilized to analyze the surface morphology of the *Mangifera indica* L. leaf powder. The sample, coated with gold, was placed on copper stubs using adhesive tape, and micrographs were obtained. The images were observed at a magnification of 1200× and 2000× using an acceleration voltage of 20.0 kV (Kashyap et al. 2023).

### 2.6 Statistical Analysis

Microsoft Excel, 2019 (Microsoft Corp., Redmond, WA) was utilized to calculate the mean and standard deviation (SD). The significant difference between the samples was determined using a one‐way ANOVA test, and the comparison between mean values was obtained by the CD (critical difference) value (Shashikant et al. 2022).

## 3 Results and Discussion

### 3.1 Proximate Analysis

Proximate analysis of the *Mangifera indica* L. leaf powder was performed to determine its nutritional composition, such as moisture content, protein, fat, ash, crude fiber, and carbohydrates. This study shows the proximate composition of *Mangifera indica* L. leaf powder in Table 1. The moisture content is a significant factor in determining the product's shelf stability. A high moisture content suggests that the product is more susceptible to microorganisms, whereas a low moisture level indicates more resistance to degradation (Princwill‐Ogbonna et al. 2019; İncili et al. 2025). Therefore, it is essential to determine the moisture content when producing value‐added products containing food ingredients. The moisture content of the mango leaf powder was determined to be 11.46% ± 0.50%. Lowering the moisture content is critical for extending storage time, delaying bacterial growth, and retarding the degradation of the product (Jaglan et al. 2023; Princwill‐Ogbonna et al. 2019). Furthermore, the ash content of the sample powder was found to be 7.56% ± 0.40%, the fat content was 4.53% ± 0.35%, and the crude fiber content was 8.50% ± 0.30%. Crude fiber is essential for promoting a healthy digestive system, encouraging regular bowel movements, and maintaining gut health. Adequate fiber intake is also associated to a reduced risk of several chronic diseases, including cancer hypercholesterolemia, type 2 diabetes, coronary heart disease, and other cardiovascular diseases (Thilagavathi et al. 2020; Usunobun et al. 2015). The protein content of the sample powder was determined to be 18.16% ± 0.76%, which suggested that the mango leaf powder is a good source of protein. The human body requires proper amounts of proteins for the replacement of damaged tissues, energy supply, and the formation of the necessary amino acids (Jaglan et al. 2023; Ogidi et al. 2019). Plants with high protein content help the body to build and repair tissues, regulate body functions, and produce essential hormones, digestive enzymes, and antibodies that help the body fight against infection (Usunobun et al. 2015). The carbohydrates in the *Mangifera indica* L. leaf powder were reported to be 49.76% ± 0.30%. The primary class of naturally occurring organic molecules known as carbohydrates is essential not only to sustain and support plants and animals' lives but also serves as a key source of raw materials for numerous industries such as food, paper, chemical, textile, and pharmaceutical. Plant carbohydrates, together with fats and proteins, are the major energy sources of food (Usunobun et al. 2015; Aborisade et al. 2017).

**TABLE 1:** Proximate composition of *Mangifera indica* L. leaf powder.

Parameters	Values (%)
Moisture content	11.46 ± 0.50
Ash	7.56 ± 0.40
Fat	4.53 ± 0.35
Crude fiber	8.50 ± 0.30
Protein	18.16 ± 0.76
Carbohydrates	49.76 ± 0.30

*Note:* Data are presented as mean ± SD (*n* = 3).

In a similar study reported by Princwill‐Ogbonna et al. (2019), the moisture content of the *Mangifera indica* L. leaf powder was found to be 20.10% ± 0.90%, ash content was 8.24% ± 0.99%, fat content was 4.30% ± 0.95%, crude fiber was 10.60% ± 0.95%, while protein and carbohydrate contents were 16.25% ± 1.00% and 60.61% ± 0.95%, respectively. In another study carried out by Adeonipekun et al. (2023) on the proximate analysis of mango flower powder, the moisture content was reported to be 12.21% ± 0.15%, carbohydrate content was 38.66% ± 0.41%, protein content was 7.20% ± 0.71%, crude fat was 19.50% ± 1.06%, ash content was 6.50% ± 0.35%, and crude fiber was found to be 16.14% ± 0.15%. The difference in the proximal composition could be attributed to various factors, including climate conditions, origin, plant age, soil type, soil quality, and genetic factors (Guzmán‐Maldonado et al. 2020).

### 3.2 Techno‐Functional Properties

The techno‐functional properties of the powder are generally linked with the water and oil interaction with the powder. They are also associated with chemical composition, rheological properties, and compatibility with other dietary components (Jha et al. 2021). These properties are highly beneficial in formulating and designing new foods (Kumar et al. 2022). The factors such as mean pore radius, total pore volume of powder, porosity, mean particle size, and particle size distribution significantly influence these functional properties (Jha et al. 2021). These properties play an important role in improving processing conditions and their use in developing novel food products (Awuchi et al. 2019). The techno‐functional properties of *Mangifera indica* L. leaves powder are given in Table 2.

**TABLE 2:** Techno‐functional properties of *Mangifera indica* L. leaf powder.

Properties	Values
Bulk density (g/cm^3^)	0.39 ± 0.001
Tapped density (g/cm^3^)	0.45 ± 0.004
Carr's index (CI)	12.40 ± 0.47
Hausner ratio (HR)	1.14 ± 0.01
Angle of repose (°)	32.01 ± 0.34
Water absorption index (g/g)	3.00 ± 0.05
Water solubility index (%)	19.80 ± 0.30
Foaming capacity (%)	32.07 ± 0.52
Foam stability (%)	21.86 ± 0.43
Swelling capacity (mL/g)	9.06 ± 0.61
Oil absorption capacity (g/g)	3.67 ± 0.05

*Note:* Data are presented as mean ± SD (*n* = 3).

#### 3.2.1 Bulk Density

The bulk density of powder measures its heaviness and is interlinked with the moisture content, particle structure and size, and interparticle forces. The high moisture content in the sample causes the particles to adhere to each other, which leads to an increase in the interspaces between them, resulting in a larger bulk volume (Singh, Inbaraj, et al. 2022; Singh, Rasane, et al. 2022). The bulk density can be improved by reducing the size of the sample. The bulk density of *Mangifera indica* L. leaf powder was determined to be 0.39 ± 0.001 (g/cm^3^). It is a significant factor in determining whether the powder is suitable for easy packaging and transport. It also has an impact on the package design and helps identify the suitable packaging material type (Awuchi et al. 2019).

#### 3.2.2 Tapped Density

The TD is determined by the mass‐to‐volume ratio of the powder obtained by compact packaging of particles on tapping (Amidon et al. 2009). On tapping, interparticle spaces between the particles are reduced. To determine TD, the volume of interparticle space can be varied according to pretreatment and packaging practices. The TD of the powder is one of its key quality characteristics, allowing for maximum packaging of the sample under external forces (Jha et al. 2021). The TD of mango leaf powder was found to be 0.45 ± 0.004 (g/cm^3^). Due to the pores in the larger particles being more occupied than in fine particles, samples with finer particles have lower porosity and higher TD than samples with coarse particles (Naji‐Tabasi et al. 2021).

#### 3.2.3 Carr's Index (CI)

Carr's index (CI) indicates the flowability of powder and is linked with bulk and TD. The value of CI < 15 indicates good flowability, whereas CI > 25 indicates poor flowability (Ribeiro et al. 2020). The Carr's index of *Mangifera indica* L. leaf powder was 12.40 ± 0.47, which indicates that mango leaf powder has good flowability. Materials with good flowability are ideal for mixing, transport, and production processes (Abe‐Inge et al. 2018).

#### 3.2.4 Hausner Ratio (HR)

The Hausner ratio (HR) determines the compressibility and flow properties of the sample. The value of HR < 1.18 indicates good flow properties, while HR > 1.35 indicates bad flow properties (Rao et al. 2021). A powder's compressibility can alter its flow properties at the microscale level due to adhesion forces between the particles (Jha et al. 2021). The Hausner ratio of *Mangifera indica* L. leaf powder was observed to be 1.14 ± 0.01, which showed that the powder has good flowability and compressibility, making it ideal for developing food products.

#### 3.2.5 Angle of Repose

The angle of repose is a parameter linked to interparticle friction during particle movement, which depends on the particles' size, shape, surface texture, and composition (He et al. 2020). It measures the powder's cohesiveness. The fluidity of the powder improves with decreasing angle of repose (Kalman 2021). The angle of repose between 25° and 30° indicates excellent powder flowability, while values over 45° indicate poor flowability (Schlick‐Hasper et al. 2022). The angle of repose of mango leaf powder was obtained to be 32.01 ± 0.34 (°); therefore, mango leaf powder has good flowability.

#### 3.2.6 Water Absorption Index and Water Solubility Index

The water absorption index (WAI) is the capacity of the powder to bind with water molecules. The WAI and WSI of *Mangifera indica* L. leaf powder were observed to be 3.00 ± 0.05 (g/g) and 19.80 ± 0.30 (%), respectively. Due to the polar or charged side chains, the high carbohydrate, fiber, and protein content is likely to promote a strong hydrogen bonding, which increases the water absorption index (Shafi et al. 2016; Sahan et al. 2015). In contrast, a low water absorption index is due to excess lipid content, which prevents the hydration of starch granules with hydrophobic components. In addition, WAI can be reduced by large particle sizes (Kraithong et al. 2018). The presence of soluble fibers and sugars influences the water solubility index. The presence of a high content of soluble fibers gives a high solubility index, while higher starch content gives a low solubility index (Sahan et al. 2015).

#### 3.2.7 Foaming Capacity and Foam Stability

These properties are influenced by various factors, including pH, viscosity, surface tension, and processing methods (Sadh et al. 2018). Foam improves the consistency, texture, and appearance of the food. The *Mangifera indica* L. leaf powder showed 32.07% ± 0.52% foaming capacity and 21.86% ± 0.43% foam stability. The main component responsible for foaming is proteins. The good foaming capacity and foam stability are linked to the powder's high protein content. In contrast, low foaming capacity is associated with globular proteins that show resistance to surface denaturation (Kumar and Saini 2016). The powder with good foaming capacity is considered a good foaming agent for making food products (Singh, Inbaraj, et al. 2022; Singh, Rasane, et al. 2022).

#### 3.2.8 Swelling Capacity

The starch's capacity to absorb water and swell is measured by the swelling capacity, also known as the swelling index, which also reflects the strength of the associative forces present in the starch granules (Awuchi et al. 2019). It represents noncovalent bonding among the starch molecules and depends on the particle size, unit operations, and processing methods (Iwe et al. 2016). The swelling capacity of *Mangifera indica* L. leaf powder was obtained to be 9.06 ± 0.61 (mL/g). Protein and starch content are frequently associated with swelling capacity. The starch granules may be trapped inside a solid protein matrix due to the higher protein content in the powder sample, which limits the starch's contact with water and ability to swell (Jha et al. 2021).

#### 3.2.9 Oil Absorption Capacity

Oil absorption capacity (OAC) is known as the binding of proteins' nonpolar side chains to the fat molecules. Foods having high protein have a high rate of oil absorption (Awuchi et al. 2019). The oil absorption capacity is linked to various factors, including particle size, thickness, overall charge density, hydrophobic nature of powder, drying methods, chemical structure, and surface properties (Sahni and Shere 2017). OAC is strongly correlated with particle size; the smaller particle size increases the oil absorption capacity (Lucas‐González et al. 2017). The oil absorption capacity of mango leaf powder was determined to be 3.67 ± 0.05 (g/g). The main component that influences the oil absorption capacity is a protein, which is made up of hydrophobic and hydrophilic parts (Shafi et al. 2016). The presence of a smaller number of amino acids can be responsible for the low oil absorption capacity of the powder (Rao et al. 2021).

### 3.3 Characterization Studies

#### 3.3.1 FTIR Analysis

**FIGURE 1:**
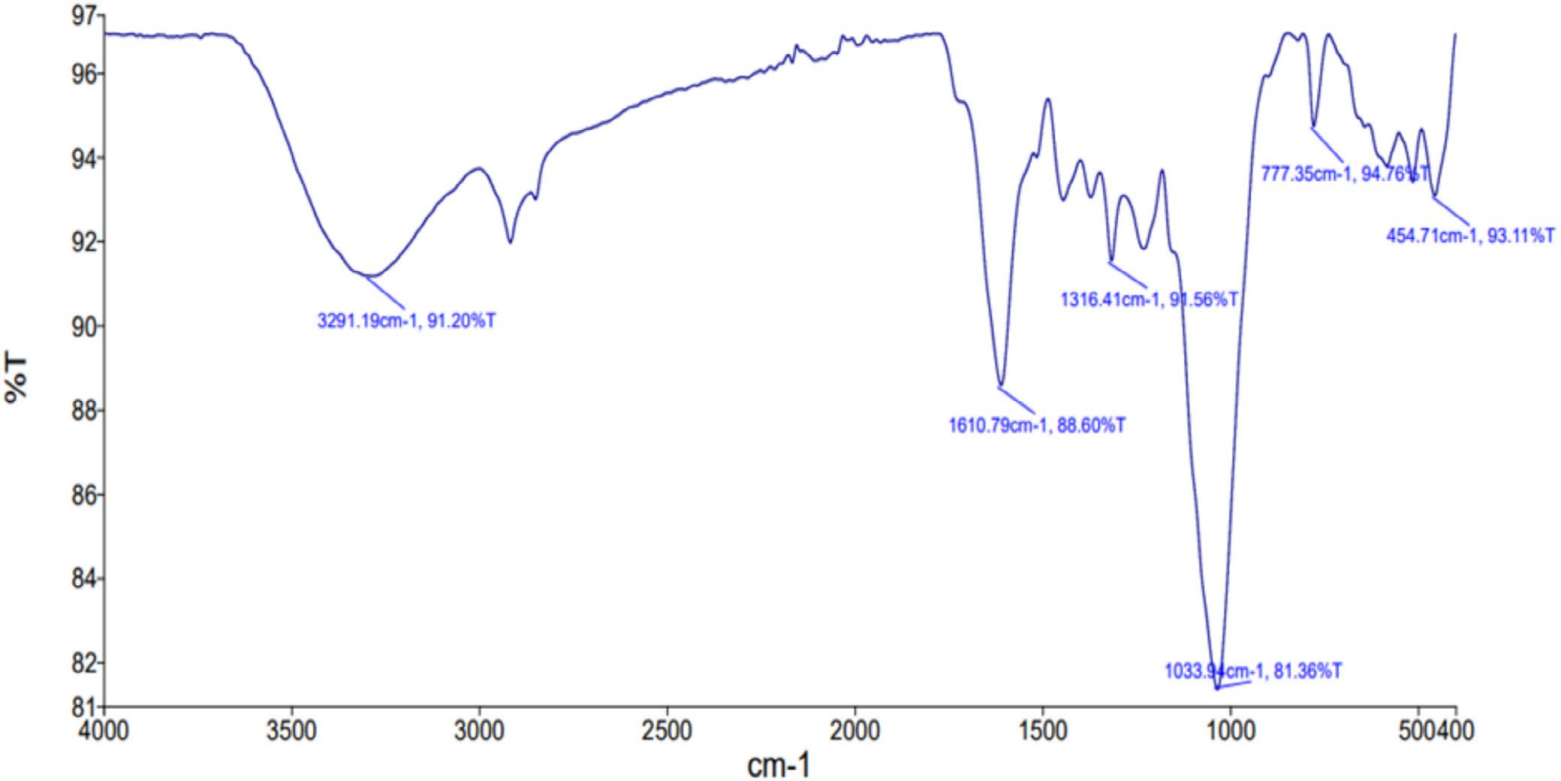
FTIR spectra of *Mangifera indica* L. leaf powder.

FTIR is employed to identify the presence and the nature of functional elemental groups, including their characteristic bond vibrations in the sample (Gong et al. 2024; Khan et al. 2019). The different peaks indicate the presence of different chemical groups in the sample (Dey et al. 2022). The resulting data provide insight into the polysaccharides' chemical composition and functional properties. In the present study, the FTIR spectra of mango leaf powder were recorded at a spectrum range of 4000–400 cm^−1^ (Figure 1). According to the spectra, the peaks were detected at 3291.19, 1610.79, 1316.41, 1033.94, 773.35, and 454.71 cm^−1^. The FTIR absorption peak at 3291.19 cm^−1^ indicated O‐H group stretching caused by intra‐ and intermolecular hydrogen bonding of alcohols or phenols present in lignin, cellulose, pectin, and hemicelluloses (Adelaja et al. 2019). The peak located at 1610.79 cm^−1^ was linked to the carbonyl group (C=O) stretching in the carboxylate structure or carboxylic acid (Khan et al. 2019; Adelaja et al. 2019). Furthermore, the peak at 1316.41 cm^−1^ indicated the C—N stretching of aromatic amines or symmetric bending of the —CH_3_ group. The peaks at 1033.94 and 773.35 cm^−1^ revealed the C—N stretching of the aliphatic amines and =C—H bending of alkenes, respectively, while the symmetric bending of the SO_4_ group occurred at 454.71 cm^−1^. The results were in accordance with the study of Adelaja et al. (2019), which revealed similar peaks in mango leaf powder at wavelengths ranging from 453.29 to 3408.33 cm^−1^.

#### 3.3.2 Thermogravimetric Analysis (TGA)

**FIGURE 2:**
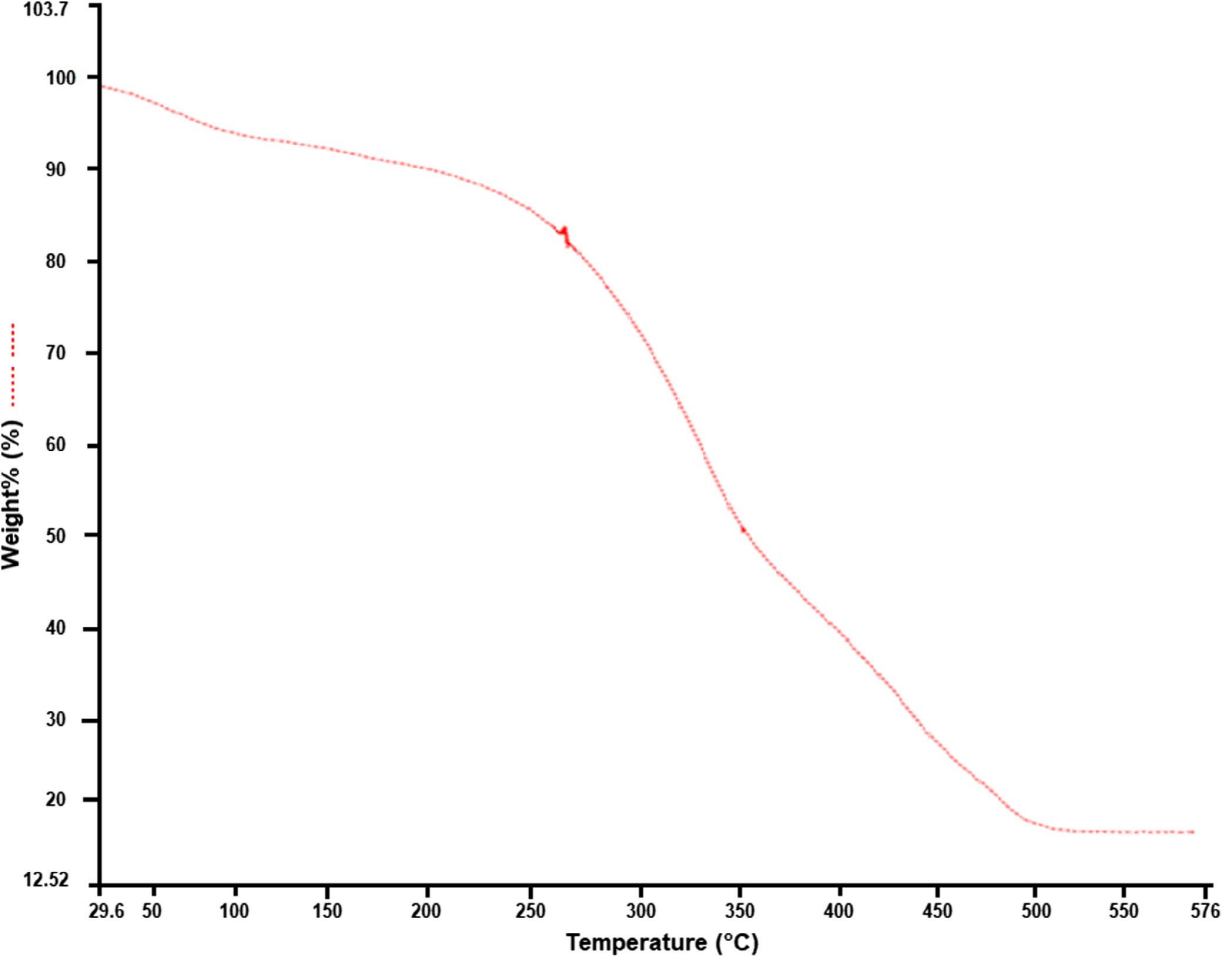
TGA of *Mangifera indica* L. leaf powder.

This technique is used to determine the thermal behavior of the powder, which measures the sample mass changes as a function of time or temperature while exposing the sample to a controlled heating environment (Junior et al. 2024; Manju et al. 2021). This method is commonly used to study the material decomposition, determination of thermal stability, degradation temperature, and residual weight of a sample (Alemán et al. 2021). In this study, *Mangifera indica* L. leaf powder was subjected to a temperature gradient of 30°C to 600°C, and the sample was heated at a rate of 10°C per minute in a nitrogen environment. The sample was kept inside a TGA instrument, and the weight of the sample was continually recorded in relation to time or temperature. The TGA data obtained from the analysis was used to generate a thermogravimetric (TG) curve (Figure 2), which displayed the sample mass loss with temperature increase. The temperature ranges of the three stages were 30°C–150°C, 225°C–400°C, and 400°C–550°C. In the first stage, the weight loss was reported to be 6.80% of the initial weight of the sample, occurred between 30°C and 150°C, and the sample mass was reduced from 100% to 93.2%, indicating the evaporation of volatile compounds and physically absorbed water present in the sample (Khan et al. 2019). The protein molecules are generally not affected at this temperature because the breakdown of proteins usually initiates at 225°C (Liu et al. 2015). In the second stage, a significant weight loss (48.04%) from 87% to 39% of the mass was reported between 225°C and 400°C, indicating the degradation of primary lignocellulosic components like hemicelluloses. The maximum mass loss in the sample occurred during this stage, and the residual mass weight was around 45% (Corazzari et al. 2015). In the third stage of degradation, between 400°C and 550°C, a 20.77% weight loss in the sample was reported, and the mass was reduced from 38% to 17.23%, indicating the degradation of secondary lignocellulosic components such as cellulose and further, the lignin degradation occurred after 480°C to 500°C (Akhtar et al. 2016; Hidayat et al. 2018). These results were comparable to the findings reported by Khan et al. (2019) and Abdullah et al. (2020) on the mango ( *Mangifera indica* ) leaf powder.

#### 3.3.3 X‐Ray Diffraction (XRD)

**FIGURE 3:**
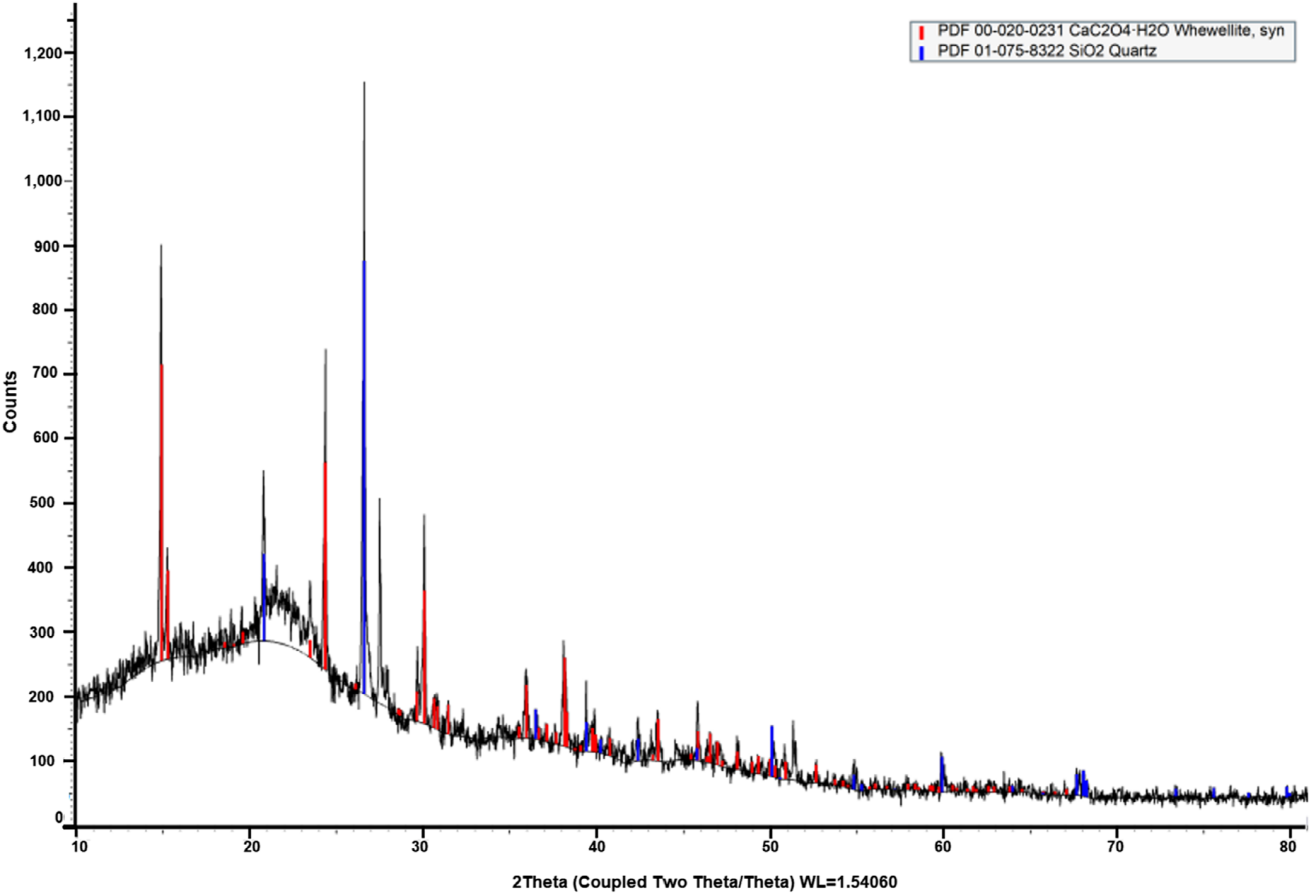
XRD of *Mangifera indica* L. leaf powder.

The XRD technique is employed to analyze the amorphous or crystalline structure of the powder as well as any changes that may have occurred during processing that may be relevant to the stability of the product (Manju et al. 2021). The orderly structure of a powder is indicated by the crystallinity index. The only crystalline component of biomass is cellulose, whereas hemicellulose and lignin are the amorphous components. A highly ordered crystalline substance typically has a number of distinct and sharp peaks, whereas an amorphous structure has a number of irregular and scattered peaks. The high solubility of amorphous structures results in their lower stability than crystalline components (Singh, Inbaraj, et al. 2022; Singh, Rasane, et al. 2022; Manju et al. 2021). The crystal structure, purity, chemical bonding, and impurities or contaminants of powder can be determined using XRD. In this study, the XRD was used to analyze the crystal structure and crystalline characteristics of *Mangifera indica* L. leaf powder (Figure 3). XRD employs X‐rays to analyze the atomic structure of a substance and examine the distance between crystal planes. The results of XRD analysis of *Mangifera indica* L. leaf powder revealed the distinct crystalline peaks at a diffraction angle (2θ) of 14.927°, 15.291°, 24.367°, 29.655°, 30.106°, 35.981°, 38.185°. It indicates that the cellulosic material has a regular lattice structure, and the OH groups present in it are bonded by strong secondary forces (Khan et al. 2019). These results were comparable with the study reported by Khan et al. (2019), which revealed the presence of crystalline peaks in the pristine mango leaf powder at 2θ values of 14.84°, 24.30°, 29.98°, and 38.08°.

#### 3.3.4 FESEM (Field Emission Scanning Electron Microscopy)

**FIGURE 4:**
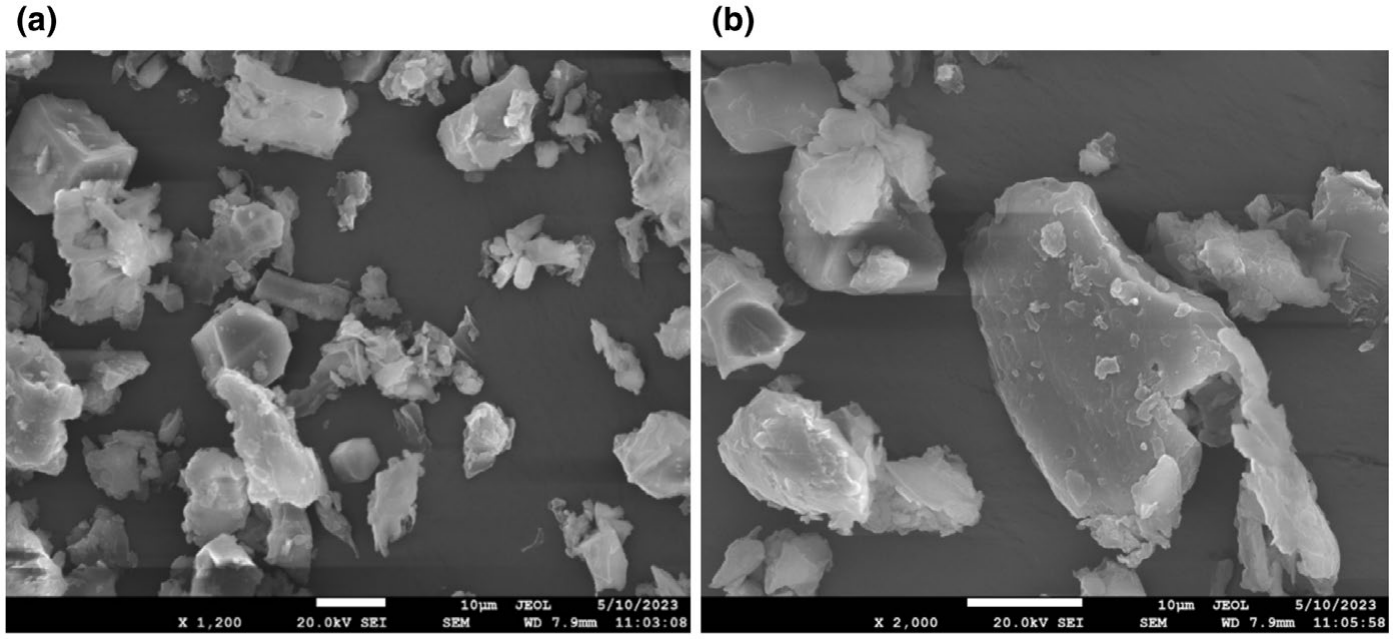
FESEM micrographs of *Mangifera indica* L. leaf powder: (a) 1200× and (b) 2000×.

FESEM is used to analyze the surface morphology and microstructure of the powder, as well as its physical properties, including particle shape, size, and distribution (Billa et al. 2024; Tosif et al. 2022). It also shows the arrangement of protein networks and starch granules in the matrix (Singh et al. 2021). In this study, the morphology of mango leaf powder was observed at a magnification of 1200× and 2000× (Figure 4). The micrographs of the mango leaf powder exhibit a rough morphology with a porous surface. The particles had indefinite shapes with an uneven surface texture (Prol et al. 2017). The micrographs were comparable with the results provided by Prol et al. (2017).

## 4 Conclusion

*Mangifera indica* L. leaf powder is a promising natural product with high nutritional value. The proximate analysis revealed that mango leaf powder is a good source of carbohydrates, fiber, and proteins. The results also reported that mango leaf powder has good techno‐functional properties, which help determine its suitability for various industrial applications, such as incorporation into food products to develop novel or functional foods, pharmaceutical drugs, cosmetic products, or herbal remedies. The characterization studies have provided valuable insight into mango leaf powder's chemical composition and structural properties. These techniques revealed the functional groups and chemical bonds present in the powder, giving details about its molecular structure. Thus, the nutritional composition, techno‐functional properties, and characterization studies of *Mangifera indica* L. leaf powder demonstrated that it can be used as a valuable ingredient in industries such as food and pharmaceuticals for developing functional foods, nutraceuticals, and pharmaceutical drugs. However, it should be emphasized that research in this area is still in progress, and more studies are needed to fully understand the possible benefits and applications of *Mangifera indica* L. leaf powder.

## Author Contributions

**Jasjit Kaur:** formal analysis (equal), investigation (equal), writing – original draft (equal). **Deepika Kaushik:** conceptualization (equal), formal analysis (equal), investigation (equal), methodology (equal), writing – review and editing (equal). **Mukul Kumar:** conceptualization (equal), formal analysis (equal), investigation (equal), methodology (equal), writing – review and editing (equal). **Gülçin Emel Babagil:** writing – review and editing (equal). **Ryszard Amarowicz:** methodology (equal). **Charalampos Proestos:** methodology (equal), writing – review and editing (equal). **Emel Oz:** writing – review and editing (equal). **Fatih Oz:** writing – review and editing (equal). **Tuba Esatbeyoglu:** writing – review and editing (equal). **Matteo Bordiga:** conceptualization (equal), writing – review and editing (equal).

## Conflicts of Interest

The authors declare no conflicts of interest.

## Data Availability

No data were used for the research described in the article.
